# Emotional symptoms and their related factors in adolescents during the acute phase of Covid-19 outbreak in South Italy

**DOI:** 10.1186/s13052-021-01036-1

**Published:** 2021-04-08

**Authors:** Simone Pisano, Gennaro Catone, Antonella Gritti, Luisa Almerico, Anna Pezzella, Pia Santangelo, Carmela Bravaccio, Raffaella Iuliano, Vincenzo Paolo Senese

**Affiliations:** 1 Department of Neuroscience, Santobono-Pausilipon Children Hospital, via Mario Fiore 6, 80129 Naples, Italy; 2 Department of Translational Medical Sciences, Federico II University, Naples, Italy; 3 Department of Educational, Psychological and Communication Sciences, Suor Orsola Benincasa University, Naples, Italy; 4 Department of Psychology, University of Campania “Luigi Vanvitelli”, Naples, Italy; 5 Neonatology Unit, “Ospedale del Mare”, Naples, Italy

**Keywords:** Pandemic, Anxiety symptoms, Depressive symptoms, Youth, Quarantine

## Abstract

**Background:**

Several studies have shown that during COVID-19 pandemic outbreak, emotional symptoms increased in the general population. Less is known about youths.

**Methods:**

We surveyed a sample of Italian adolescents during the strictest quarantine period and assessed the effects of socio-demographic and psychological factors on current emotional symptoms. A convenient sample of 326 adolescents (age range 14–19 years) participated in a web-based survey. We collected data on several socio-demographic and psychological variables (summarized into three indexes: environmental context, changes in lifestyle, and worries about infection) and psychopathological symptoms (previous psychopathological status, current anxiety and depressive symptoms).

**Results:**

Descriptive analysis showed that adolescents have experienced quarantine under very different conditions; they reported 47.5 and 14.1% of anxiety and depressive symptoms, respectively. Regression analyses indicated that previous psychopathological status and worries about infection are linked to anxiety and that female gender, previous psychopathological status (moderated by change in lifestyle), worse environmental context are linked to depression.

**Conclusion:**

This study indicates that, facing the COVID-19 pandemic and its related safety measures, adolescents show relevant emotional symptoms and therefore should be monitored, assessed and supported.

**Supplementary Information:**

The online version contains supplementary material available at 10.1186/s13052-021-01036-1.

## Background

Since March 2020, Italy has been facing the COVID-19 outbreak, soon after the outbreak in China, slightly before other world countries. The Italian government had enacted a strict quarantine in the attempt to reduce the contagion. Fear of personal and relatives’ contagion, and worries for physical health prompted social distancing and isolation. The resulting economic difficulties presented a wealth of highly distressing factors [1]. For example, it is well known that quarantine itself may produce negative psychological effects including post-traumatic stress symptoms, confusion, and anger [2]. It is likely that suicide and other dangerous behaviours may increase [1, 3]. A recent review revealed a high prevalence of psychiatric symptoms in general population during COVID-19 pandemic and pointed out that we all are still unaware of long-term outcomes [4]. There is a clear consensus among experts that mental health status during pandemic outbreaks should be assessed and mental health problems addressed [1, 5]. This is particularly true regarding fragile populations such as health workers, psychiatric patients and youths [1, 2, 6]. We here focus on the latter. Adolescents may face stressors such as fears of infection, frustration and boredom, inadequate information, lack of in-person contact with classmates, friends, and teachers, lack of personal space at home, and family financial loss to a greater degree than adults [7]. The routes to psychiatric disorders usually pass through adverse events and traumas. Thus, it is imperative to empirically study the psychopathological effects of a pandemic outbreak, such as COVID-19, and the consequences of its related safety measures, such as social distancing and quarantine. This, in fact, would allow planning of possible interventions to prevent negative psychological consequences in the event of future pandemics. In a recent study focusing the adolescent Chinese population, Zhou et al. [8] revealed a prevalence of depressive symptoms, anxiety symptoms, and a combination of depressive and anxiety symptoms during COVID-19 outbreak of 43.7, 37.4, and 31.3%, respectively, much higher than estimates usually reported in China in pre-COVID era. Another study, not limited to adolescents, but including young adults, reported that nearly 40% of youths are prone to psychological problems [9]. In Italy, a study on adults reported a high prevalence of those suffering from high or very high levels of distress, with females being more susceptible [10]. Another study on adults reported that about 38% displayed mild to severe likelihood of psychological distress, with cyclothymic, anxious and depressive temperaments being risk factors [11]. Here, we aimed at expanding these findings by providing more evidence related to adolescents, and by contextually and thoroughly assessing environmental and psychological factors that may be linked to the raise of emotional symptoms (state anxiety and depressive symptoms). We collected the data between April 25th and May 13th 2020, which strictly corresponds to the highest level of social restrictions imposed by Italian Government (full lockdown; see also [12]), in order to catch the putative psychological impact of both the strictest quarantine measures and the greatest exposure to the pandemic outbreak. We hypothesize a higher prevalence of emotional symptoms during this period compared to previously reported results. Also, based on previous studies [13, 14], we hypothesize that a previous general tendency to psychopathology, unfavourable environment, such as an uncomfortable and “not connected” quarantine living context, and acute psychological changes, such as lifestyle changes and higher worries about COVID-19, could be factors related to emotional symptoms.

## Methods

### Participants

The recruitment of participants was initiated through schools. Schools were chosen based on previous contact between them and the research team, their previous experience on epidemiological surveying, on their known compliance to mental health research and their availability in organize and expedite dissemination of the survey. Among the four schools contacted, three agreed to participate; they are placed in the metropolitan area of Naples, in Campania region, south of Italy; they all were professional institutes that, culturally, in Italy, are more frequented by males and by low/mid income families.

Before the data collection, participants’ parents and each adolescent had the opportunity to carefully read an informative sheet about the research that the schools had provided and to ask for any questions to teachers and researchers; then, parents and youths were requested to agree to participate in the study through an online informed consent form. A total of 472 subjects responded to their school’s invitation. Of these, 412 (87.3%) gave their consent to the study, and 326 (79.1%) completed the whole survey and were included in the analyses. We present data from the final sample, that included 247 males (75.8%, age range 14–19 years, *M* age = 15.8, *SD* = 1.3) and 79 females (24.2%, age range 14–19 years, *M* age = 16.0, *SD* = 1.4), excluding those who did not filled the whole survey. Males and females did not differ on age, *t* (324) = 1.20, *p* = 231. We lack data on those who did not gave their consent. The study was approved by the Ethical Committee of the “A.O.R.N. Antonio Cardarelli - A.O.R.N. Santobono-Pausilipon” Hospital of Naples (Italy).

### Procedures

The study was conducted through the Qualtrics online survey platform between April 25th and May 13th 2020. During this period, schools disseminated to their students a link to the online platform that presented the survey. The data described here are those collected cross-sectionally. The whole study is expected to be longitudinal with data collection within a 6 month period that will include longitudinal outcomes. To this end, each participant was assigned an anonymous alpha-numeric code that allow the researchers to link the answers to the follow-up data collection.

In regard to the cross-sectional data presented here, the survey included two sections: (a) a sociodemographic questionnaire (SQ) devised ad hoc and investigating the living context and quarantine conditions of participants, everyday lifestyle habits and worries about COVID-19 (see supplementary file); and (b) a section including mental health standardized measures: State-Trait Anxiety Inventory (STAI), Mood and Feelings Questionnaire-short form (MFQ-SF) and Strength and Difficulties Questionnaire (SDQ).

### Measures

#### Socio-demographic questionnaire (SQ)

A socio-demographic questionnaire asking gender, age, the characteristics of the dwelling (i.e., number of rooms, presence of private room, open spaces, web-connection availability etc.), the changes in everyday lifestyle habits related to the quarantine (e.g., sleep, feeding, etc.), and worries about COVID-19 (e.g. afraid of possible own and relative’s infection), was administered. It was created in order to capture a wide range of enviromental and psychological domains, based on the purpose of exploring protective and risk factors for short and long term psychopathology, as similarly done by other groups [14]. In particular, questions related to the characteristics of the living space and its potential comfort, the technological equipment available, the changes in lifestyle and the presence of worries for the self or the relatives linked to the infection were included. These are all potential sources of psychological distress and maladjustment that may influence how the quarantine had been lived [13, 14]. The answers collected in this section have been used to create three indexes able to measure the living context and psychological changes of the participants during the quarantine and investigate the relationship of these indexes with the supposed psychopathological effects of the pandemic: specifically, (1) environmental context (EC), is an index that summarize the responses related to the participants’ living space and its potential comfort; higher scores on this measure indicate that participants had more space available in their homes, open, accessible spaces, the availability of a good and fast web-connection as well as a good device to do so; (2) changes in lifestyle (CL), is an index that summarize the responses related to the participants’ changes in everyday lifestyle habits; higher scores on this measure indicate higher changes in the quality and quantity of feeding and sleeping habits; (3) worries about infection (WI), is an index that summarize participants’ responses related to worries about themselves and their close family members of being exposed to the COVID-19 infection; higher scores on this measure indicate that participants had higher worries and tendency to collect information about the contagion.

#### State-trait anxiety inventory (STAI)

The Italian version of State-Trait Anxiety Inventory (STAI, form Y) [15, 16] is a commonly used measure of anxiety, with good psychometric properties. The STAI form Y has 40 items, 20 items developed to measure the state anxiety (S-A) and 20 items developed to measure the trait anxiety (T-A). Items of the S-A scale assess intensity of current feelings (“at this moment”) and are rated on a 4-point scale: 1 = “not at all”, 2 = “somewhat”, 3 = “moderately”, and 4 = “very much”. Items of the T-A scale assess frequency of feelings “in general” and are rated on a 4-point scale: 1 “almost never”, 2 = “sometimes”, 3 = “often”, and 4 = “almost always”. For both scales, higher scores indicate greater anxiety, state or trait respectively. In the present paper, only the S-A was administered as a measure of current anxiety symptoms. Observed Cronbach’s alphas was .912. A value of 40 can be considered a clinical threshold value (cut-off) predictive of anxiety symptoms, distinguishing three levels of severity: mild (from 40 to 50), moderate (from 51 to 60) and severe (scores greater than 61) [17].

#### Mood and feelings questionnaire-short form (MFQ-SF)

The self-report child short form of the Mood and Feelings Questionnaire [18, 19] was used to assess current depressive symptoms. The MFQ-SF includes 13 items that cover depressive symptoms over the past 2 weeks. It is a widely used instrument for the screening of depressive symptoms in the general population of adolescents, with good psychometric properties. In the present study, the original 13-item MFQ-SF was translated into Italian by bilingual psychologists and then back-translated by two independent translators to verify the equivalence of the translated scale to the original one [20]. An exploratory factorial analysis (Principal Factor Analysis) carried out on the scale confirmed the unidimensional structure; eigenvalues (and percentage of explained variance) of the first three factors were respectively 5.76 (44.3%), 1.19 (9.2%) and 0.96 (7.4%). For the purpose of the present study, a total score was computed. Higher scores indicate greater depression. In this study, Cronbach’s alpha was .887. Angold and colleagues pointed out that, in the short version of the scale, the value of 12 can be considered as a clinical threshold value (cut-off) predictive of depressive symptomatology [18].

#### Strength and difficulties questionnaire (SDQ)

The Italian self-report version of the Strength and Difficulties Questionnaire (SDQ) [21] was administered to assess the general psychopathology. The SDQ includes 25 items, related to five domains: (1) emotional symptoms (5 items), (2) conduct problems (5 items), (3) hyperactivity-inattention problems (5 items), (4) peer problems (5 items), and (5) prosocial behaviour (5 items). Individual items are scored on a 3-point scale, 0 = “not true”; 1 = “somewhat true”; and 2 = “certainly true”, with a score range 0–10 for each subscale. The psychometric properties of the self-report version of the SDQ are generally acceptable to good across studies [22]. For the purpose of the present study, we used the total score (excluding the prosocial behaviour scale) as a measure of general psychopathology referring to the last 6 months. Higher scores indicate greater psychopathological symptoms. In this study, Cronbach’s alpha was .738. A four-band categorization of the total score was suggested: “close to the average” (from 0 to 14), “slightly raised” (from 15 to 17), “high” (from 18 to 19) and “very high” (from 20 to 40) [23].

### Statistical analyses

Descriptive statistics were firstly used to describe demographics and data related to the living context during the quarantine, the lifestyle changes and worries about COVID-19, the state anxiety (STAI S-A), the depressive symptoms (MFQ-SF) and the general psychopathology symptoms (SDQ). The frequency distribution of responses and/or means and standard deviation of variables were reported. Then, responses to items within each of the three considered domains, environmental context (EC), changes in lifestyle (CL) and worries about infection (WI), were analysed to extract a single indicator useful to summarize the several questions of the sections. Once extracted, the association between the three indicators and emotional symptoms controlling for previous general psychopathological status was investigated. To this aim, two hierarchical multiple regression analyses were carried out on the state anxiety scores (STAI S-A) and the depression scores (MFQ-SF) respectively. In both models, in the first step, the gender (dummy coded: male = 1, female = 0) and age (z-score) were included as control variables, in the second step, the SDQ score (z-score) was included as the independent variable, in the third step the three indicators (z-scores) were included as the independent variables, whereas in the final fourth step, the two-way interaction effects were considered to test the moderation effect of the three stress indicators on the relation between the SDQ and the considered dependent variables.

## Results

### Socio-demographic section

#### Environmental context (EC)

Analyses of responses collected in this section of the protocol showed that, with regard to the place of residence (item 1), 73.6% of the participants experienced quarantine in a dwelling with four or more rooms (excluding bathrooms and closets, *M* = 4.3, *SD* = 1.3). Most of the adolescents did not have a room for themselves (item 2), but shared their own room with a brother/sister (53.4%), while 32.5% had their own room to study and sleep in. With regard to open spaces (item 3), 3.7% had no outdoor space available in the house (only windows) or in a condominium, 5% had only a condominium space available (courtyard or green area), 46.3% had a balcony, while 45.1% had a terrace or garden. Regarding the possibility to access the Internet (item 4), the data showed that 3.1% did not have access to the Internet, 9.5% accessed the Internet with their mobile phone or for a limited time, 3.1% accessed the Internet via Wi-Fi but for a limited time, 58.6% accessed the Internet via unlimited time Wi-Fi, while 25.8% accessed the Internet via fast and unlimited time Wi-Fi. With regard to the availability of devices to connect to the Internet (item 5), the data showed that 33.7% had a mobile phone only, 3.1% had a tablet, 36.2% a shared PC and 27.0% a PC for exclusive use. To get a single measure of environmental context (variable named EC), each of the five items of the section were recorded into three levels of comfort (low, medium and high) and a principal component analysis (PCA) was carried out by extracting one single component and computing the factor score. The PCA showed that the unidimensional solution explained 30.7% of the variance, with each item showing a saturation > .497. The higher was the score on this index, the better was the living context during the lockdown.

#### Changes in lifestyle (CL)

Analyses of responses collected in this section of the protocol showed that there were changes in both eating habits and sleep-wake rhythms during the quarantine. In particular, with regard to eating habits, the data showed that 82% of adolescents stated that they had modified their diet from a quantitative point of view (item 1, 54.0% “a little”, 28.2% “a lot”). 57.9% stated that they had modified their diet from a qualitative point of view (item 2, 42.9% “a little”, 15.0% “a lot”). 68.4% declared to have modified the times (hours and frequency) of alimentation (item 3, 43.2% “a little”, 25.2% “a lot”). With regard to the analysis of changes in biological rhythms and sleep quality, more generalized changes emerged. In fact, 65.0% of adolescents declared that they had modified “a lot” the sleep-wake rhythms (item 4), 29.4% “a little”, while only 5.5% “not at all”. With regard to the quality of sleep (item 5), 40.5% of adolescents reported that they had modified the quality of sleep “very much”, 37.7% “a little”, while only 21.8% “not at all”. To get a single measure of changes in lifestyle (variable named CL), a principal component analysis (PCA) was carried out on the five items of this section by extracting one single component and computing the factor score. The PCA showed that the unidimensional solution explained 44.2% of the variance, with each item showing a saturation > .594. The higher was the score on this index, the higher were the changes in the lifestyle during the lockdown.

#### Worries about infection (WI)

Analyses of responses collected in this section of the protocol showed that, during the quarantine, adolescents were more worried about their families getting infected (item 1), *M* = 7.2, *SD* = 3.1, than they were worried about themselves (item 2), *M* = 4.3, *SD* = 3.6; the comparison between these two scores is significant, *t* (325) = 14.71, *p* < .001. In particular, 38.0% declared they were not worried about contracting the virus themselves, while only 10% reported they were not worried about a family member contracting the virus. In the latter case, 31% declared a maximum level of concern, i.e. “10”. Finally, with regard to the time spent reading or listening to information related to contagion (item 3), the majority of adolescents (50%) reported they spent about 1 h, 13.2% spent two or more hours, while 12.9% do not know anything at all. To get a single measure of worries about infection (variable named WI), a principal component analysis (PCA) was carried out on the three items of the sections by extracting one single component and computing the factor score. The PCA showed that the unidimensional solution explained 53.1% of the variance, with each item showing a saturation > .589. The higher the score on this index, the higher were the worries about the infection during the lockdown.

### Psychopathology section

#### State anxiety

The assessment of state anxiety symptoms during the COVID-19 revealed that the adolescents had a mean score of 41.6 (*SD* = 10.8); considering the cut-off of 40 as predictive of clinically relevant symptoms [17], data showed that the 47.5% of the sample exceeded it; specifically, 27.0% showed “mild anxiety”, 14.1% showed “moderate anxiety” and 6.4 “severe anxiety”. A significant gender difference was observed, *t* (324) = 5.74, *p* < .001, with females showing higher state-anxiety (S-A) than males (see Table 1).

**Table 1:** Descriptive statistics of the main variables as a function of the gender

	Females			Males			Total sample		
Variable	*M*	*SD*	% above cut-off	*M*	*SD*	% above cut-off	*M*	*SD*	% above cut-off
Age	16.0	1.4	−	15.8	1.3	−	15.9	1.3	−
SDQ	14.6	6.2	46.8	10.4	5.4	20.2	11.4	5.9	26.7
STAI S-A	47.4	11.9	70.9	39.8	9.7	40.1	41.6	10.8	47.5
MFQ-SF	10.1	6.3	29.1	5.4	4.9	9.3	6.5	5.6	14.1

#### Depression

The assessment of depressive symptoms during the COVID-19 revealed that adolescents had a mean score of 6.5 (*SD* = 5.6); considering the cut-off of 12 as predictive of clinically relevant symptoms [18], data showed that 14.1% of the sample exceeded it. A significant gender difference was observed, *t* (324) = 6.89, *p* < .001, with females showing higher depression (MFQ-SF) than males (see Table 1).

#### General psychopathology

The assessment of the presence of general psychopathology symptoms referred to the 6 months (thus before the onset of pandemic) showed that adolescents had a mean total score of 11.4 (*SD* = 5.9); considering a cut-off score of 14, data indicate that 26.7% of the sample exceeded it; specifically, 9.2% showed a “slightly raised” score, 6.1% showed a “high” score, 11.3% showed a “very high” score. A significant gender difference was observed, *t* (324) = 5.80, *p* < .001, with females showing more symptoms (SDQ) than males (see Table 1).

### Relationship between extracted indicators (EC, CL and WI) and emotional symptoms

Data from the hierarchical regression analysis are reported in Table 2. Results showed a similar pattern of effects for the two considered dependent variables. In regard to state anxiety, data showed that over and above the control variables (gender and age), the general psychopathology symptoms (SDQ) were uniquely associated with the anxiety scores, *R*^2^_diff_ = .294, *p* < .001. As expected, the model-fit increased when the three indicators were entered into the model, *R*^2^_diff_ = .019, *p* = .017, whereas the last step did not show significant two-way interaction effects. The parameters of the final model revealed that general psychopathology symptoms (SDQ), *β* = .556, *p* < .001 and worries about infection (WI), *β* = .110, *p* = .013 were both uniquely independent predictors of anxiety, *R*^2^ = .425, *p* < .001. No other significant effects were observed. That is, over and above the other variables in the model, the higher the general psychopathology symptoms before the COVID-19 and the worries about the infection, the higher the state anxiety during the quarantine was.

**Table 2:** Results of the regression analysis (final models) exploring the impact of hypothesized explanatory variables on emotional symptoms

	State Anxiety			Depression		
Variable^	*b*	*beta*	*p-value*	*b*	*beta*	*p-value*
Males	−0.198	−0.085	0.067	−0.239	−0.103	0.012
Age	0.077	0.077	0.074	0.016	0.016	0.672
SDQ	0.556	0.556	0.000	0.625	0.625	0.000
EC	0.010	0.010	0.810	−0.106	−0.106	0.005
CL	0.082	0.082	0.065	0.108	0.108	0.006
WI	0.110	0.110	0.013	0.044	0.044	0.254
EC × SDQ	−	−	−	−0.067	−0.066	0.086
CL × SDQ	−	−	−	0.138	0.139	0.000
WI × SDQ	−	−	−	0.003	0.003	0.928

*Note*. *N* = 326; ^Males = Gender of participants (dummy coded: male = 1, female = 0); Age = Age of participants (years; z-score); SDQ = Strength and Difficulties Questionnaire total score (z-score); *EC* Environmental context index (z-score), *CL* changes in lifestyle index (z-score), *WI* Worries about infection index (z-score)

**Fig. 1:**
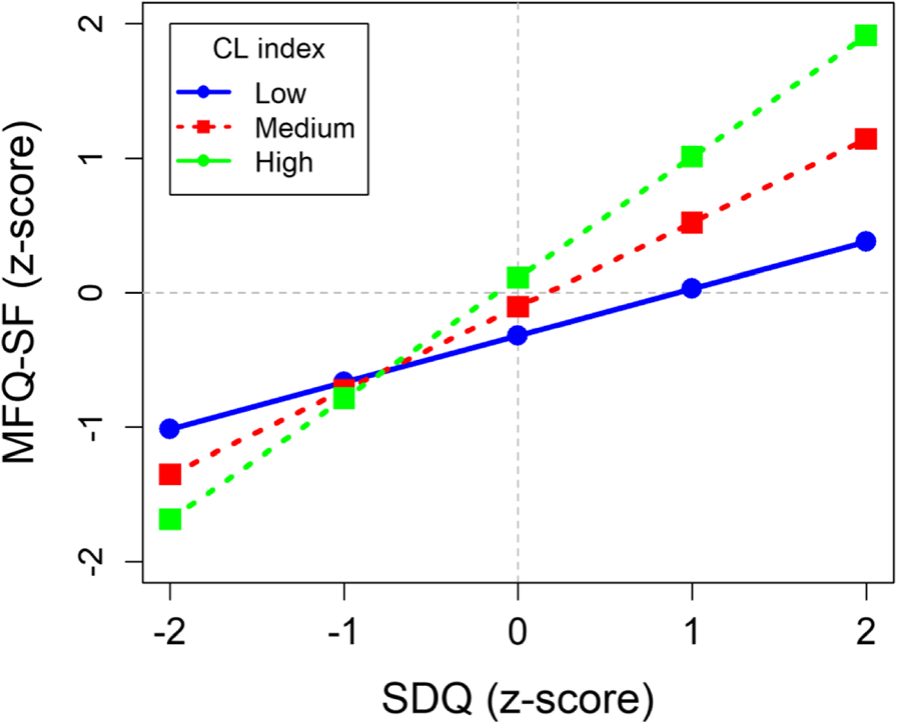
Example of moderation effect of the CL index on the association between SDQ and the MFQ-SF. To depict the figure, the other consider variables of the model (Male, Age, EC and WI) were fixed to their zero value

In regard to depression, data showed that over and above the control variables (gender and age), the general psychopathology symptoms (SDQ) were uniquely associated with the depression scores, *R*^2^_diff_ = .390, *p* < .001. As expected, the model-fit increased when the three stress indicators were entered into the model, *R*^2^_diff_ = .020, *p* = .003, whereas in this model, also the last step showed significant two-way interaction effects between indicators and the general psychopathology, *R*^2^_diff_ = .023, *p* < .001. The parameters of the final model revealed that gender, *β* = −.103, *p* = .012, general psychopathology symptoms (SDQ), *β* = .625, *p* < .001, environmental context (EC), *β* = −.106, *p* = .005, and changes in lifestyle (CL), *β* = .108, *p* = .006 were all uniquely independent predictors of depression, *R*^2^ = .569, *p* < .001, and that the amount of changes in lifestyle (CL) moderated the relation between the general psychopathology and the depression scores (see Fig. 1). No other two-way interaction effects were observed. That is, over and above the other variables in the model, females showed a higher level of depression than males, the more were the general psychopathology symptoms before the COVID-19 the higher was the depression during the quarantine; also, the better the living context the lower the depressive symptoms were, the higher the changes in lifestyle the more the depressive symptoms were; finally, the combination of the general psychopathology and changes in lifestyle increased the depressive symptoms. Indeed, the relationship between general psychopathology and depression was stronger for those who reported having changed their lifestyle habits a lot than for those who report having changed their lifestyle habits a little or not at all.

## Discussion

We here assessed emotional symptoms and their related factors in adolescents during the strict quarantine period due to COVID-19 pandemic in Italy. We found a prevalence of anxiety and depressive symptoms of respectively 47.5 and 14.1% of the sample. These estimates frankly exceeded the previously reported ones in Italy, assessed in a context without any pandemic, via two previous epidemiological studies; in fact, Frigerio et al. [24] found a prevalence of internalizing disorders of 6.5% and Gritti et al. [25] found a prevalence of internalizing symptoms (in clinical range) of 10.5%. Compared to other studies performed during quarantine period in China, estimates are closer, although with some notable differences. Regarding anxiety, Zhou et al. [8] found very similar percentages (43.7%), whereas regarding depression their values were higher (37.4%). Cultural aspects or differences in measurement instruments and/or sample sizes may account for this discrepancy. Compared to studies on the general population, using as reference estimates reported in Xiong et al. [4] who summarize the most reliable ones (using those reported by better appraised studies, e.g. those with the most representative samples), as well as a recent large survey on Italian general adult population [26], ours are higher for anxiety and similar for depression (in their review anxiety symptoms range from 6.3 to 18.7% and depressive symptoms range from 14.6 to 32.8% [4]; similarly, Fiorillo reported 12.4% of depressive symptoms and 17.6% of anxiety symptoms [26]). Globally, our findings add evidence of the enhanced need of psychological and psychiatric care for adolescents during the health emergency; more specifically, although in need of replication with further data, the notion that adolescents are a fragile population, more prone to anxiety symptoms than adults, is confirmed. Efforts to mitigate psychological burden should be a priority for policymakers and health organizers.

We extend our investigations to factors related to emotional symptoms. We wanted to be as inclusive as possible extracting several empirical factors that maybe be theoretically linked to anxiety and depression [13] and assessing previous psychopathological status with a standardized measure (SDQ). The results of our regression analysis showed that more general psychopathological symptoms and higher worries for infection predict heightened anxiety; whereas that female gender, more general psychopathological symptoms, worse environmental quarantine context and changes in lifestyle habits (moderated by psychopathological symptoms) predict heightened depression. Thus, we confirm that subjects suffering from previous psychopathological symptoms are more prone to suffer from an increase of emotional symptoms during the pandemic [26]; more in depth, worries for infection (own and relative’s infection) are linked to anxiety symptoms, whereas subjects, especially females, living in unfavourable environmental context and who experienced changes in lifestyle habits are more prone to suffer from depression. Highlighting these characteristics seems important to direct more intensive and more tailored prevention and treatment strategies to the right targets. Social, socio-economic and health support should be firstly directed to people who meet these characteristics.

The study presents some limitations that should be highlighted. First of all, the sample is relatively small, skewed towards males and we lack a rigorous epidemiological sampling methodology. This because we wanted to gather data, with adequately powered sample size, during the acute phase of pandemic outbreak and when the quarantine was strictest, thus the short window of available time prevented a wider dissemination of the online survey. The study then lacked a proper epidemiological sampling of the schools. Those that accepted to participate in the study were all professional institutes, that, as already said, in our region, are mostly representative of low-medium socio-economic conditions, with a higher male prevalence. This is reflected by the low number of females in our sample and the lack of objective data on socio economic status; accordingly, firm conclusions on gender differences cannot be drawn and the generalizability to the general population is limited. Our socio demographic questionnaire was designed to summarize data on living context, but not on the overall socio-economic status, which may, but also may not, correspond. Other studies need to cross evaluate these two factors to draw a finest picture of the impact of socio economic and environmental factors on pandemic-related psychopathology. Also, as all the data were self-reported and rely on an online survey, our study may suffer from social desirability bias or other sampling biases. Ideally, multi informant surveys and/or cross check with personal assessment should be performed to gather data less prone to such bias, but this was impossible during a quarantine period. Of course, our study is cross sectional in nature and thus conclusion on causality should be confirmed with longitudinal ones. Based on previous studies, we focused our study on internalizing symptoms, but it is likely that externalizing ones increase as well; future studies should assess both and their interplay.

Notwithstanding these relevant limitations, although in need of replication and expansion, we can provide useful data to be translated for a better services organization and more targeted policy decisions. Adolescents need to be supported and protected with any efforts during the sanitary emergency due to COVID-19; emotional symptoms are substantially higher compared to a “normal” period and have to be monitored, assessed and detected; those with previous psychopathological symptoms are even more fragile and in need of social and health support.

## Supplementary Information

**Additional file 1.** Socio-demographic questionnaire (SQ).

## Data Availability

The datasets used and/or analysed during the current study are available from the corresponding author on reasonable request.

## Abbreviations

SQ: Socio demographic questionnaire
STAI: State-Trait Anxiety Inventory
MFQ-SF: Mood and Feelings Questionnaire-short form
SDQ: Strength and Difficulties Questionnaire
EC: Environmental context
CL: Changes in lifestyle
WI: Worries about infection
