# Supplementary material for: Emotional symptoms and their related factors in adolescents during the acute phase of Covid-19 outbreak in South Italy
Source: Ital J Pediatr. 2021 Apr 8;47:86. doi: 10.1186/s13052-021-01036-1 (PMC8026329; doi:10.1186/s13052-021-01036-1)
Supplement: Supplementary file 1 — Additional file 1. Socio-demographic questionnaire (SQ). [file 13052_2021_1036_MOESM1_ESM.doc]

**MANUSCRIPT TITLE**: Emotional Symptoms and their Related Factors in Adolescents During COVID-19 Outbreak in Italy

**SUPPLEMENTARY MATERIALS**

**SOCIO-DEMOGRAPHIC QUESTIONNAIRE (SQ)**

**SECTION 1: Environmental Context (EC)**

**Item 1.1**: How many rooms are there in the house where you are living (excluding bathrooms and closets)?

**Item 1.2**: In the house where you are living, do you have a room where you can stay alone?

🞏 Yes, I have my own room where I sleep and study

🞏 Yes, I have a room where I study alone but it's not only mine

🞏 No, I share my room to study and sleep with my brother / sister

🞏 No, I don't have my own room in the house

**Item 1.3**: In the house where you are living, do you have an outdoor space available? (you can select more options)

🞏 Garden (large)

🞏 Garden (small)

🞏 Terrace (large)

🞏 Terrace (small)

🞏 Balcony (large)

🞏 Balcony (small)

🞏 Common courtyard

🞏 Condominium park

🞏 Only windows

**Item 1.4**: During this period, which of these devices do you have available? (You can select more options)

🞏 A tablet that I mainly use

🞏 A tablet that I share with others

🞏 A computer (PC) that I mainly use

🞏 A computer (PC) that I share with others

🞏 My personal cell phone

**Item 1.5**: In the house where you live, do you have Internet available? (You can select multiple options)

🞏 Yes, I have unlimited access with a very fast Wi-fi

🞏 Yes, I have unlimited access with normal Wi-fi

🞏 Yes, I have Wi-fi access but for a limited time

🞏 Yes, I log in using my mobile phone

🞏 Yes, but little time per day

🞏 I don't have internet at home

**SECTION 2: Changes in Lifestyle (CL)**

*Now you will read some statements about your daily habits and biological rhythms. To each statement answer by indicating how true it is for you. There are no right or wrong answers. To answer, use the following choices: "not at all", "moderately", "totally".*

**Item 2.1**: In these last two weeks, I have changed my feeding from a quantitative point of view (e.g. I eat more or less)

**Item 2.2**: In these last two weeks, I have changed my feeding from a qualitative point of view (e.g. I eat different foods, I drink different drinks

**Item 2.3**: In these last two weeks, I have changed my feeding times (e.g. I eat at different times, more or less frequently etc.)

**Item 2.4**: In these last two weeks, I have changed my sleep-wake rhythms (e.g. I go to bed or wake up earlier or later)

**Item 2.5**: In these last two weeks, the quality of my sleep has changed (e.g. I sleep better or worse)

**SECTION 3: Worries about Infection (WI)**

**Item 3.1a**: Do you feel worried that one of your family members may contract coronavirus (COVID-19)?

🞏 Yes

🞏 No

**Item 3.1b**: Using a scale from 1 (= little) to 10 (very much), indicate how afraid you are that one of your family members will contract the Coronavirus (COVID-19)

**Item 3.2a**: Are you worried about the possibility of contracting the Coronavirus (COVID-19)?

🞏 Yes

🞏 No

**Item 3.2b**: Using a scale from 1 (= little) to 10 (very much), indicate how afraid are you of contracting the Coronavirus (COVID-19)

**Item 3.3a**: Do you inform yourself about the Coronavirus (COVID-19)?

🞏 Yes

🞏 No

**Item 3.3b**: Indicate in general how much time (in hours) do you spend reading or listening to news related to the infection of the Coronavirus (COVID-19).
